# Efficient on-chip terahertz generation and detection with GaN photoconductive emitters

**DOI:** 10.1038/s41377-025-01870-6

**Published:** 2025-06-25

**Authors:** Can B. Uzundal, Qixin Feng, Weichen Tang, Chen Hu, Collin Sanborn, Yoseob Yoon, Sudi Chen, Jiawei Ruan, Steven G. Louie, Feng Wang

**Affiliations:** 1 Department of Physics, University of California, Berkeley, CA USA; 2 Materials Sciences Division, Lawrence Berkeley National Laboratory, Berkeley, CA USA; 3 Department of Chemistry, University of California, Berkeley, CA USA; 4 Graduate Group in Applied Science and Technology, University of California, Berkeley, CA USA; 5 Department of Mechanical and Industrial Engineering, Northeastern University, Boston, MA USA; 6 Kavli Energy NanoScience Institute, Berkeley, CA USA

**Keywords:** Terahertz optics, Nonlinear optics, Ultrafast photonics, Photonic devices

## Abstract

Photoconductive emitters for terahertz generation hold promise for highly efficient down-conversion of optical photons because it is not constrained by the Manley-Rowe relation. Existing terahertz photoconductive devices, however, faces limits in efficiency due to the semiconductor properties of commonly used GaAs materials. Here, we demonstrate that large bandgap semiconductor GaN, characterized by its high breakdown electric field, facilitates the highly efficient generation of terahertz waves in a coplanar stripline waveguide. Towards this goal, we investigated the excitonic contribution to the electro-optic response of GaN under static electric field both through experiments and first-principles calculations, revealing a robust excitonic Stark shift. Using this electro-optic effect, we developed a novel ultraviolet pump-probe spectroscopy for in-situ characterization of the terahertz electric field strength generated by the GaN photoconductive emitter. Our findings show that terahertz power scales quadratically with optical excitation power and applied electric field over a broad parameter range. We achieved an optical-to-terahertz conversion efficiency approaching 100% within the 0.03–1 THz bandwidth at the highest bias field (116 kV/cm) in our experiment. Further optimization of GaN-based terahertz generation devices could achieve even greater optical-to-terahertz conversion efficiencies.

## Introduction

Terahertz technology is expected to play an increasingly important role in ultrahigh bandwidth communication^1^, non-intrusive medical and security imaging^2^, and quantum materials spectroscopy^3–6^. However, its widespread adoption is hindered by the current lack of efficient, high-power terahertz sources. Despite significant advancements in terahertz devices, further progress in both terahertz detection and generation requires novel approaches and materials^7^.

For terahertz generation, femtosecond lasers have been widely used as the driving source through two nonlinear optical processes: optical rectification (i.e. difference frequency generation) and photoconductive emitters (i.e. the Auston switch^8^)^9–12^. In terms of the optical power conversion efficiency ($\eta =\frac{{P}_{\text{THz}}}{{P}_{\text{optical}}})$, optical rectification is fundamentally limited by the Manley-Rowe relation: even with a quantum conversion efficiency of 100%, the terahertz power conversion efficiency is lower than 1% due to the very low terahertz photon energy (4 meV) compared with near infrared photons (1.0–1.5 eV)^13^. The optical power conversion efficiency of photoconductive emitters on the other hand, is not limited by the Manley-Rowe relation. Photoconductive emitters exploit the creation of free carriers in a semiconductor upon absorption. Under the influence of an external electric field the photogenerated carriers are accelerated, leading to a transient surge current inside the medium. In this case, the far-field radiation is proportional to the time derivative of the transient surge current, similar to a dipole antenna^14^. In principle, terahertz generation with an optical-to-terahertz power conversion efficiency beyond 100% is possible with photoconductive emitters as the terahertz radiation gains power from the capacitive electrical energy stored ($U=\frac{1}{2}C{V}^{2}$, where C is the capacitance of the emitter, V is the applied DC bias) in the absence of light^14,15^.

To realize these high efficiencies experimentally, however, is challenging. The highest optical-to-terahertz power conversion efficiency reported in the literature is ~7.5% using a photoconductive emitter based on low-temperature (LT) GaAs^16^, the material of choice in existing terahertz emitter technology. To reach the 7.5% power conversion efficiency, great effort has been made to optimize the nanoantenna design^15–21^. Further improvement in terahertz generation efficiency has been stalled due to fundamental limitations of the already optimized LT-GaAs material.

Qualitatively, the terahertz power generated by a photoconductive emitter scales quadratically with the transient current as ${P}_{\text{THz}}\propto {j}^{2}=j\,\cdot\, {ne}{v}_{\text{sat}}$. Here *j* is the maximum transient current density, *n* is the photoexcited carrier density, *v*_*sat*_ is the saturation velocity reached under high bias electric field and we use the well-established Drude-like description of transient current $j={ne}{v}_{\text{sat}}$^14^. The optical excitation power is ${P}_{{\rm{opt}}}\propto n\hslash {\omega }_{{\rm{opt}}}$. The optical-to-terahertz conversion efficiency therefore scales as $\eta =\frac{{P}_{\text{THz}}}{{P}_{\text{opt}}}\propto \frac{e}{\hslash {\omega }_{\text{opt}}}\,\cdot\,j\,\cdot\, {v}_{\text{sat}}$. The maximum transient current is constrained by the saturation excitation density where the screening electrical field approaches the bias electric field *E*_bias_. In principle, a higher *E*_bias_ can increase the maximum achievable transient current *j* and therefore the terahertz generation efficiency^12^, but the value of *E*_bias_ is fundamentally limited by the breakdown electric field *E*_br_ of the semiconductor. Considering these factors, in terms of intrinsic properties of the photoconductive material, the optical-to-terahertz conversion efficiency can scale as $\eta \propto \frac{e}{\hslash {\omega }_{\text{opt}}}\,\cdot\,{E}_{\text{br}}\,\cdot\,{v}_{\text{sat}}$. This scaling suggests that dramatic improvement of the terahertz generation efficiency can be achieved by using semiconducting materials with a high breakdown electric field and saturation velocity. The wide bandgap Gallium Nitride (GaN) features a breakdown electric field of 3 MV/cm (over 10 times higher than low temperature grown (LT) -GaAs^22^) and a saturation electron velocity of 2 × 10^7 ^cm/s^23^ (over 2 times higher than LT-GaAs^24^). Therefore, GaN-based photoconductive emitters could enable more than an order of magnitude improvement of terahertz conversion efficiency compared with emitters based on GaAs materials simply from scaling analysis. Yet, experimentally GaN-based photoconductive emitters have been so far little explored^25,26^.

Here we demonstrate high terahertz generation efficiency with on-chip GaN-based photoconductive emitters. We use an ultraviolet femtosecond pump laser to excite a transient photocurrent in a biased semi-insulating (si) GaN emitter which launches a terahertz pulse into a coplanar waveguide. We track the transient terahertz field in the coplanar waveguide using a pump-probe spectroscopy technique that uses the excitonic electro-optic response of GaN. We show that the terahertz emission power scales quadratically with the bias electric field and the pump intensity over a broad parameter range. The highest sustainable bias electric field in our device is between 100–200 kV/cm. Photoinduced electrical damage gradually takes place at higher biases. The optical-to-terahertz conversion efficiency in the GaN photoconductive emitter can reach as high as 58% within the 0.1 to 1 THz and close to 100% in the 0.03 to 1 THz bandwidth. Significantly higher terahertz generation efficiency could be anticipated in optimized GaN photoconductive emitters where an electric field approaching the intrinsic breakdown limit can be applied.

## Results

### Steady state device characteristics

**Fig. 1:**
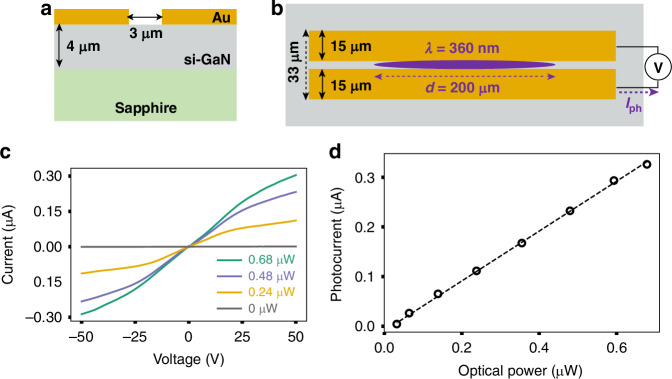
Steady state device characteristics **a** Cross section of the devices with planar 60 nm thick Au contacts on a 4-µm-thick si-GaN substrate grown on sapphire. **b** A schematic illustration (see Fig.S1a for a microscope image) of a co-planar stripline device with a 3 µm photoconductive gap. The stripline is illuminated with an elliptical UV pulse (purple ellipse) (f_rep_ =75.6 kHz, λ = 360 nm, t_pulse_ = 250 fs) which drives the photocurrent (I_ph_) under an applied bias (V). **c** Current-voltage characteristics of the device under illumination at 4 different incident optical powers. **d** Photocurrent at 50 V as a function of optical power shows a linear dependence

We fabricate planar electrical contacts on a thin film GaN substrate using standard photolithography processes. Figure 1a illustrates the device cross section. The contacts form a coplanar stripline as depicted in Fig. 1b. We evaluate the photocarrier generation and collection efficiency of the coplanar stripline through photoconductivity measurements. The device shows a dark resistance of 250 GΩ, which decreases upon UV illumination due to photo-induced carriers. With a UV excitation power of 0.6 µW (λ = 360 nm, *f*_rep_ = 75.6 kHz), the resistance drops to approximately 100 MΩ (Fig. 1c). Figure 1d demonstrates that the photocurrent increases linearly with laser excitation power under a bias of 50 V. From the photocurrent data, we estimate a responsivity (*R*_*measured*_) of 0.5 A/W. Assuming each incident photon (λ = 360 nm) corresponds to a collected electron, we estimate the responsivity at unity quantum yield (*R*_*unity*_) as 0.29 A/W. We define photoconductive gain $G={R}_{measured}/{R}_{unity}$^27,28^ and obtain *G* = 1.7 for our device, indicating an efficient collection of photocarriers.

Steady-state photoconductivity measurements suggest efficient generation and collection of carriers. Under femtosecond excitation, the photocurrent in GaN can vary at the picosecond time scale. Such ultrafast dynamics and carrier transport of photoexcited GaN have not been previously established. To gain a fundamental understanding of terahertz generation from GaN photoconductive emitters, it is essential to quantitatively characterize the transient terahertz pulse with sub-picosecond time resolution. Towards this objective, we first investigated the static electro-optic properties of GaN and then developed a novel ultrafast UV pump-probe spectroscopy to measure the transient electric field in situ using the electro-optic response of GaN.

### Excitonic electro-optic effect

**Fig. 2:**
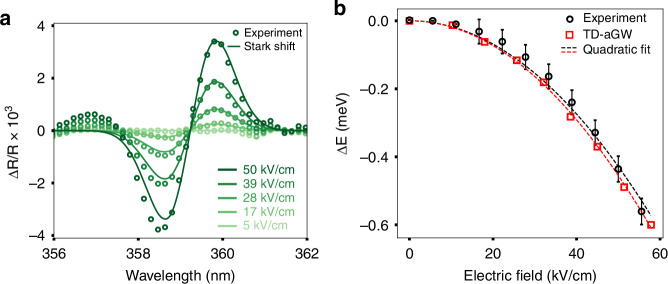
Excitonic Electro-optic Effect in GaN. **a** Electro-optic spectrum of si-GaN measured under electric fields ranging from 0 to 50 kV/cm. The darker shade of green indicates larger electric fields. **b** The shift in the central energy of the exciton, ∆E = E_ex_(F) – E_ex_(F = 0), as a function of electric field, F, shows a quadratic dependence for both the experiment (black dashed lines and circles) and the numerical calculations (red dashed lines and squares). The details of the numerical method are provided in SM-3

We characterize the electro-optic response of GaN by applying an electric field (*F*) and measuring the reflectance contrast ($\frac{\Delta R}{R}$) at a given wavelength (*λ*). The reflectance contrast is defined as the change in reflectance of GaN under electric field, normalized with respect to the reflectance in the absence of electric field ($\frac{\Delta R}{R}=\frac{R\left(F,\lambda \right)-R\left(F=0,\lambda \right)}{R\left(F=0,\lambda \right)}$). In Fig. 2a, we report the reflectance contrast spectrum around the band edge of GaN, showing a prominent feature which we assign to the red shift of the exciton resonance of GaN under electric field. The reflectance spectrum of si-GaN supports the exciton picture as we observe a corresponding reflectance peak around the same photon energy (*E*_exciton_ = 3.451 eV) (Fig. S1b). A robust excitonic effect in GaN can exist at room temperature because of its large exciton binding energy (*E*_b_ = 20.4 meV). Two excitons with similar binding energies, known as A & B excitons, are present in GaN. They appear as a single combined peak at room temperature due to thermal broadening^29,30^. Similar electro-optic effects in GaN have been previously observed in electro-absorption measurements^31–33^, yet the physical details of the excitonic electro-optic effect and its dependence on static electric field was not previously investigated.

Figure 2a also depicts the electric-field dependence of the reflectance contrast spectrum. The amplitude of the reflectance contrast increases quadratically with the DC-field. Employing a fitting procedure (see SM-1 for details), we extract the field-dependent shift of the combined exciton resonance (*ΔE*). Figure 2b reports a redshift of 0.56 ± 0.04 meV at *F* = 60 kV/cm. The red shift and its uncertainty correspond to an electric field sensitivity of 2 kV/cm (at a 5σ confidence level). The quadratic red shift of the exciton resonance is attributed to the DC Stark effect where the Stark shift is $\varDelta E=-\frac{1}{2}\alpha {F}^{2}$ and *α* is the polarizability^34^. Using this expression, we experimentally estimate the effective exciton polarizability of GaN as *α*_exp_ = 3.4 × 10^−10 ^cm^2 ^meV/V^2^.

The absence of a linear Stark Shift and the predominance of the quadratic Stark effect in the case of GaN is expected from GaN’s crystal field symmetry. We present the symmetry analysis in SM-2 and conclude that the quadratic Stark shift is the only symmetry allowed contribution. We further verify our understanding by performing first-principles calculations and determine the effect of the applied electric field in the presence of excitonic absorption. We outline the details of the ab-initio method in SM-3. In our ab-initio approach we include a dephasing factor of 5.8 meV which simulates the experimental spectral broadening. Consistent with the experimental results, our first-principles calculations also predict a quadratic red shift of the exciton (Fig. 2b, red squares, see also Fig. S2a) while the polarizability obtained from our numerical simulations (Fig. 2b, red dashed lines) *α*_th_ = 3.6 × 10^−10 ^cm^2 ^meV/V^2^ shows excellent agreement with the experimental value.

### Transient electric field measurements

**Fig. 3:**
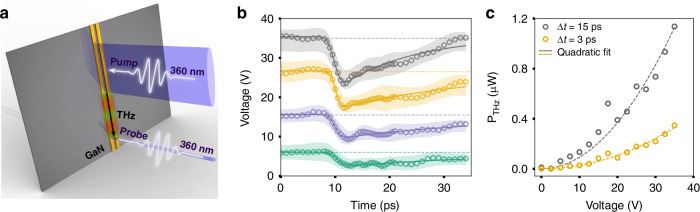
Transient electric field measurements. **a** Schematic illustration of the ultrafast approach that employs the exciton Stark effect to investigate the terahertz pulses that propagate along the waveguide. **b** Terahertz pulses generated using 1 µW pump at 4 different biases, measured using the scheme outlined in (**a**). The voltage traces, V(t), start at the applied bias and maintains this value until terahertz pulse arrives at the probe location at around *t* = 8 ps which initiates a voltage drop. The shaded regions around each time trace correspond to the uncertainty in the field measurements estimated using run-to-run variations in the data. **c** Using the V(t) traces shown in (**b**), the average terahertz power is calculated for the initial 3 ps (yellow) or 15 ps (gray) of the pulse. The corresponding η is measured as 34% within the first 3 ps of the pulse and η = 113% within the first 15 ps of the pulse

Figure 3a illustrates the ultrafast scheme we developed to quantify the transient terahertz electric field in GaN using the excitonic Stark effect. Our method is akin to electro-optic sampling where the terahertz field induced changes to the refractive index of the parent material is measured by an optical probe pulse. In our approach, a femtosecond excitation pulse (pump) excites free carriers and generates a transient current (*j*) in the biased coplanar stripline. The transient current launches a propagating terahertz wave along the stripline. The stripline supports a waveguide mode that allows the terahertz wave to propagate with negligible loss^35^. A second femtosecond beam (probe) with a controllable time delay measures the terahertz wave at a separate location along the waveguide using the excitonic Stark effect. Because the pump and probe beams are spatially well separated, the pump-induced change in the probe reflection is only due to the transient terahertz wave. Analyzing the change in the terahertz pulse arrival time as a function of pump-probe separation reveals the effective refractive index of the terahertz waveguide mode as 2.45 (Fig. S3).

### Evaluation of terahertz generation efficiency

In Fig. 3b, we present calibrated transient voltage traces (*V*(*t*)) for select bias voltages (*V*_*i*_) when the absorbed pump power is 1 µW (i.e. *P*_optical_ = 1 µW). The shaded region around each voltage-time trace represents the uncertainty in our time-domain measurement which is on the order of ±2 V. The voltage trace exhibits a swift negative drop characterized by a time constant *τ*_a_, followed by a gradual recovery characterized by the charge depletion with a time constant *τ*_r_. Both *τ*_a_ and *τ*_r_ can be extracted using a phenomenological fitting function (see SM4 for details). Under low bias (i.e., *V*_*i*_ < 20 V) charge depletion time constant *τ*_r_ is sluggish (*τ*_*r*_ > 20 ps); however, it becomes significantly faster at high bias, reaching *τ*_*r*_ = 12 ps at *V*_*i*_ = 35 V (Fig. S4a). Meanwhile, *τ*_*a*_ remains constant at around 1–2 ps (Fig. S4b), which is comparable to the propagation time of the terahertz pulse across the pump spot size (1.6 ps assuming 200 µm spot size with *n*_eff_ = 2.45) and puts an upper bound on the bandwidth of the measured terahertz pulses. The terahertz pulse bandwidth can be improved with shorter pump pulse duration and pump spot size.

We quantify the average terahertz power in the waveguide (*P*_THz_) and the optical-to-terahertz conversion efficiency ($\eta =\frac{{P}_{\text{THz}}}{{P}_{\text{optical}}}$) using the *V*(*t*) traces (Fig. 3b). The instantaneous power in the waveguide is $P\left(t\right)=\frac{{\left({V}_{i}-V\left(t\right)\right)}^{2}}{{Z}_{0}}$, where *Z*_0_ is the characteristic impedance of the waveguide. We estimate *Z*_0_ = 70 Ω using the analytical formulas for a coplanar stripline waveguide on a dielectric^36^. Numerical integration of the instantaneous power over a time window (Δ*t*) gives an estimate of the terahertz pulse energy, ${E}_{\text{pulse}}=\mathop{\int }\nolimits_{t=0}^{\varDelta t}P\left(t\right){dt}$. The average terahertz power is ${P}_{\text{THz}}={E}_{\text{pulse}}{f}_{\text{rep}}$ where *f*_rep_ is the repetition rate of the optical source. As shown in Fig. 3c, the terahertz power in the waveguide scales quadratically with the applied bias. At the highest sustainable bias voltage *V*_bias_ of 35 V, the terahertz radiation has a power of 0.35 µW when integrated over the first 3 ps, corresponding to a 35% of the optical-to-terahertz power conversion efficiency. Extending the integration time window to 15 ps, the optical-to-terahertz conversion efficiency can reach 113% at *V*_bias_ = 35 V, which corresponds to a bias electric field of 116 kV/cm.

**Fig. 4:**
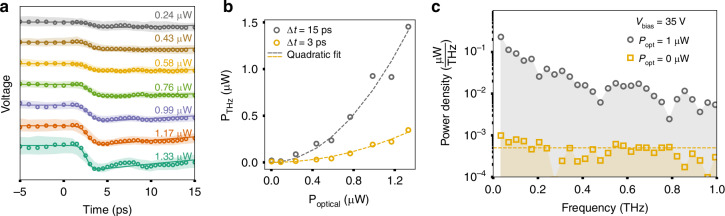
Terahertz generation characteristics of strip-line waveguides on GaN. **a** Terahertz pulse traces at V_bias_ = 35 V measured as a function of absorbed pump intensity. Traces are each offset by 10 V for clarity. The shaded regions around each time trace correspond to the uncertainty in the field measurements estimated using run-to-run variations in the data. **b** The average terahertz power in the pulse as a function of absorbed intensity in 3 ps (yellow) and 15 ps of the pulse (gray). **c** Power density spectrum at ***P***_optical_ = 1 µW and V_bias_ = 35 V (gray circles). The noise floor is experimentally determined using measurements at ***P***_optical_ = 0 µW and V_bias_ = 35 V (yellow squares)

We further show the pump power dependence of *V*(*t*) at a fixed *V*_bias_ = 35 V in Fig. 4a. The terahertz transient signal becomes stronger at higher pump power. Figure 4b shows the average terahertz radiation power as a function of the absorbed optical excitation power. The yellow and gray symbols are the average terahertz power when integrated over the initial 3 ps and 15 ps of the terahertz pulse, respectively. Under both integration time windows, terahertz radiation power scales quadratically with the absorbed optical excitation power in the experimental range. Using Fourier transformation of a 30 ps long scan, we can obtain the terahertz power density as a function of frequency at *P*_optical_ = 1 µW and *V*_bias_ = 35 V (Fig. 4c). We obtain a terahertz radiation power of 0.58 µW over 0.1–1 THz and 0.92 µW over 0.03–1 THz, corresponding to an optical-to-terahertz power conversion efficiency of 58% and 92% in the respective bandwidths.

## Discussions

Here we have demonstrated efficient terahertz generation within a waveguide using wide bandgap GaN based photoconductive emitters. The waveguided terahertz pulse can be readily radiated into free space by connecting the waveguide to a suitable broadband antenna. Such GaN-based emitters already represent a five to tenfold improvement in optical-to-terahertz conversion efficiency compared to the state-of-the-art LT-GaAs based emitters^16^. Moreover, the quadratic scaling of terahertz power in terms of electric field implies that GaN photoconductive emitters could achieve even higher efficiency if stronger bias electric field can be used. Currently, our planar device’s performance is limited by gradual laser-induced damage at electric fields exceeding 100-200 kV/cm and fluences on the order of 0.1–1 GW/cm^2^. This sustained electric field under intense optical illumination is already higher than those typical for GaAs-based photoconductive emitter devices^37–39^, yet it is still one order magnitude lower than GaN’s intrinsic breakdown field of 3 MV/cm. GaN-based devices capable of operating closer to the intrinsic breakdown field have been realized in GaN power electronics using optimized device configurations^40–42^. We envision that optimized GaN terahertz generation devices operating close to the intrinsic breakdown field could realize highly efficient terahertz sources for a wide variety of applications. For instance, high power terahertz emitters can be fabricated using arrays of GaN emitters. When illuminated, these arrays produce a single coherent terahertz pulse, with power proportional to the size of the array^43^.

## Methods

### Device fabrication and characterization

The waveguide-integrated terahertz photoconductive switches were fabricated on a si-GaN thin film on sapphire substrate (Fe compensated with doping concentration of 10^19 ^cm^−3^ and resistivity >10^7^ Ohm-cm, MSE Supplies, USA). We first thoroughly cleaned the GaN chips with acid piranha solution (3 parts concentrated H_2_SO_4_ 1 part 30% H_2_O_2_). Then the chips were dipped in 3:1 mixture of concentrated HCl and water to remove the surface oxide layer. 6 nm Ni and 60 nm Au are patterned on the sample through standard double-layer photolithography, sputtering and liftoff processes. We used a Keithley 2502 Picoammeter to characterize the DC characteristics of the fabricated devices.

In section SM5 of the supplementary information, we provide an extensive qualitative discussion of factors that may influence both the electric field dependent redshift of the exciton feature and the terahertz generation results. Additionally, we present terahertz data from a different, second device (Fig. S5), measured one month after the original experiments described in the main text. These results reproduce the time trace from the main text within the experimental uncertainty.

### Ultrafast UV pump-probe spectroscopy

We operated an amplified Yb:KGW fs-laser (Light Conversion Carbide) at a repetition rate of 75.6 kHz as the pump laser for a commercial optical parametric amplifier (OPA, Light Conversion Orpheus). Carbide provided 200 fs long pulses at a central wavelength of 1030 nm with 4 W average power. Meanwhile, the signal output of the OPA, tuned to a central wavelength of 720 nm with an average power of 0.13 W was used as the fundamental beam. The fundamental beam was frequency doubled (SHG) in a β-BaB_2_O_4_ (β-BBO) crystal using a type-I phase matching geometry. SHG efficiency was optimized by angle tuning the BBO crystal and was 11%. Using a UV bandpass filter (Edmund Optics, 12090), the UV light was separated from the remaining fundamental. The UV beam (λ = 360 nm) was split into a pump and a probe beam using a non-polarizing beamsplitter. The pump beam polarization and intensity were adjusted using a half wave plate followed by a polarizer. The pump polarization (V in the laboratory frame) was chosen to be perpendicular to the probe polarization (H polarization) in all the experiments. Using a cylindrical lens on a rotation mount and an adjustable iris, the pump beam was shaped into a light sheet of variable size and orientation after a 10× microscope objective. For the experiments in the manuscript, the pump beam was 200 by 5 µm. The pump and probe beams were recombined using another beamsplitter and imaged on to the sample using a microscope objective (10×, 0.30 NA). The reflected pump was blocked using a beam block while letting the probe beam through. Further, an α-BBO polarizer (Thorlabs GLB10-UV) was used to separate any residual pump beam after the beam block. We detected the probe beam using a UV-enhanced photodiode. The diode photocurrent was amplified using a current amplifier (Ithaco 1211) and sent through a lock-in amplifier (SRS830) for detection. For terahertz measurements, the photoconductive switch was biased with a sine wave (*f* = 209 Hz) using a function generator (Rigol, DG1022Z) connected to a voltage amplifier (PiezoDrive TD250). The lock-in amplifier was configured to detect the 2 *f* of the bias sine wave.

As a function of time delay between the pump and probe pulses, we record the reflectance contrast of the probe at a given bias which constitutes our time dependent signal, *S(t)*. To convert *S(t)* into the *V(t)* traces presented in Figs. 3 and 4, we obtain a look up table of the reflectance contrast as a function of bias, *S(V)*, by sweeping the applied voltage in the absence of pump light. Using this look up table we convert *S(t)* into *V(t)*. As a result, *V(t)* presented throughout the main text, always starts at the applied voltage and only shows transient behavior when the pump pulse arrives.

Throughout the manuscript we reference *P*_optical_ as the absorbed optical power, which we obtain by measuring the incident optical power with a calibrated power head, we define this quantity as *P*_*incident*_. We correct *P*_*incident*_ for the Fresnel loss at the GaN-air interface assuming (n = 3.1 at 360 nm see Fig. S1e, reflectivity R = 73.8% assuming normal incidence) and a geometrical factor, Y, which accounts for the larger width of the pump beam compared to the width of the photoconductive gap (Y = 3/4.5 = 0.66). Therefore, the reported *P*_*optical*_ = 0.5*P*_*incident*_.

### Static electro-optic spectrum

The ultrafast UV pulses, generated as described above, has a wide bandwidth. For the static measurements outlined in Fig. 2, we insert a monochromator (Acton Research, 275, 600 grooves/mm) and select a narrow UV wavelength range to investigate. By tuning the signal output of the OPA between 700 nm to 740 nm and accordingly adjusting the phase matching angle of the β-BBO we obtain a tunable narrow band UV source between 350 nm to 370 nm. Then by scanning the grating and recording the reflectance contrast at each wavelength, we construct the spectra shown in Fig. 2.

### Theoretical simulations

First-principles calculations of the electronic structure of GaN were performed using DFT-PBE in Quantum ESPRESSO^44,45^, with a plane-wave energy cutoff of 100 Ry and experimental lattice constants (*a* = 3.19 Å, *c* = 5.19 Å). The *GW* (*G*_*0*_*W*_*0*_ level) and *GW*-BSE calculations for quasiparticle and optical properties were done with BerkeleyGW^46^, using a dielectric cutoff of 40 Ry, a 9 × 9 × 6 grid, and 1600 bands. The Hybertsen-Louie plasmon-pole model was used for dynamical screening^47^. For BSE calculations, the interaction kernel was interpolated to a finer grid around the Γ-point equivalent to 48 × 48 × 33 grid in the whole Brillouin zone, and transitions between 3 valence and 1 conduction band were included to ensure convergence.

The electro-optic effect of GaN was computed using the TD-a*GW* approach^48^, with real-time propagation of the density matrix under an external light field. The time-dependent interacting density matrix was computed with the TD-a*GW* Hamiltonian, incorporating equilibrium quasiparticle energies, light-matter interactions, and excitonic effects. A dephasing factor of 5.8 meV was used to simulate spectral broadening.

Absorption spectra under static electric fields were calculated using a “pump-probe” setup^49^, where the dielectric function was derived from the Fourier transforms of the probe field and induced polarization. Time propagation was done with a 0.0048 fs step using the fourth order Runge-Kutta method, and a time window of *T* = 3840 fs for Fourier transformations was applied. Three valence and one conduction band were included in the simulations with a patched *k*-grid around the Γ-point equivalent to 48 × 48 × 33 grid in the whole Brillouin zone.

## Supplementary information

Supplementary Material to Efficient On-Chip Terahertz Generation and Detection with GaN Photoconductive Emitters
