# Supplementary material for: Efficient on-chip terahertz generation and detection with GaN photoconductive emitters
Source: Light Sci Appl. 2025 Jun 25;14:226. doi: 10.1038/s41377-025-01870-6 (PMC12198356; doi:10.1038/s41377-025-01870-6)
Supplement: Supplementary file 1 — Supplementary Material to Efficient On-Chip Terahertz Generation and Detection with GaN Photoconductive Emitters [file 41377_2025_1870_MOESM1_ESM.docx]

**Supplementary Material**

**Efficient On-Chip Terahertz Generation and Detection with GaN Photoconductive Emitters**

Can B. Uzundal^1,2,3*^, Qixin Feng^1,2*^, Weichen Tang^1,2^, Chen Hu^1,2^, Collin Sanborn^2,4^, Yoseob Yoon^1,2,5^, Sudi Chen^1,2^, Jiawei Ruan^1,2^, Steven G. Louie^1,2^ and Feng Wang^1,2,6^

^1^Department of Physics, University of California, Berkeley, CA, USA

^2^Materials Sciences Division, Lawrence Berkeley National Laboratory, Berkeley, CA, USA

^3^Department of Chemistry, University of California, Berkeley, CA, USA

^4^Graduate Group in Applied Science and Technology, University of California, Berkeley, CA, USA

^5^Department of Mechanical and Industrial Engineering, Northeastern University, Boston, MA, USA

^6^Kavli Energy NanoScience Institute, Berkeley, CA, USA

*equal contribution

e-mails: [fengwang76@berkeley.edu](mailto:fengwang76@berkeley.edu)

**Contents**

[SM1-Electro-optic spectra of si-GaN 1](#_Toc187949448)

[SM2-Crystal symmetry constraints on the excitonic Stark effect 4](#_Toc187949449)

[SM3-Details of the numerical method 6](#_Toc187949450)

[SM4-Pump-probe curve fitting and distance dependence 9](#_Toc187949451)

[SM5-Reproducibility of results 11](#_Toc187949452)

[SM6-References: 14](#_Toc187949453)

SM1-Electro-optic spectra of si-GaN


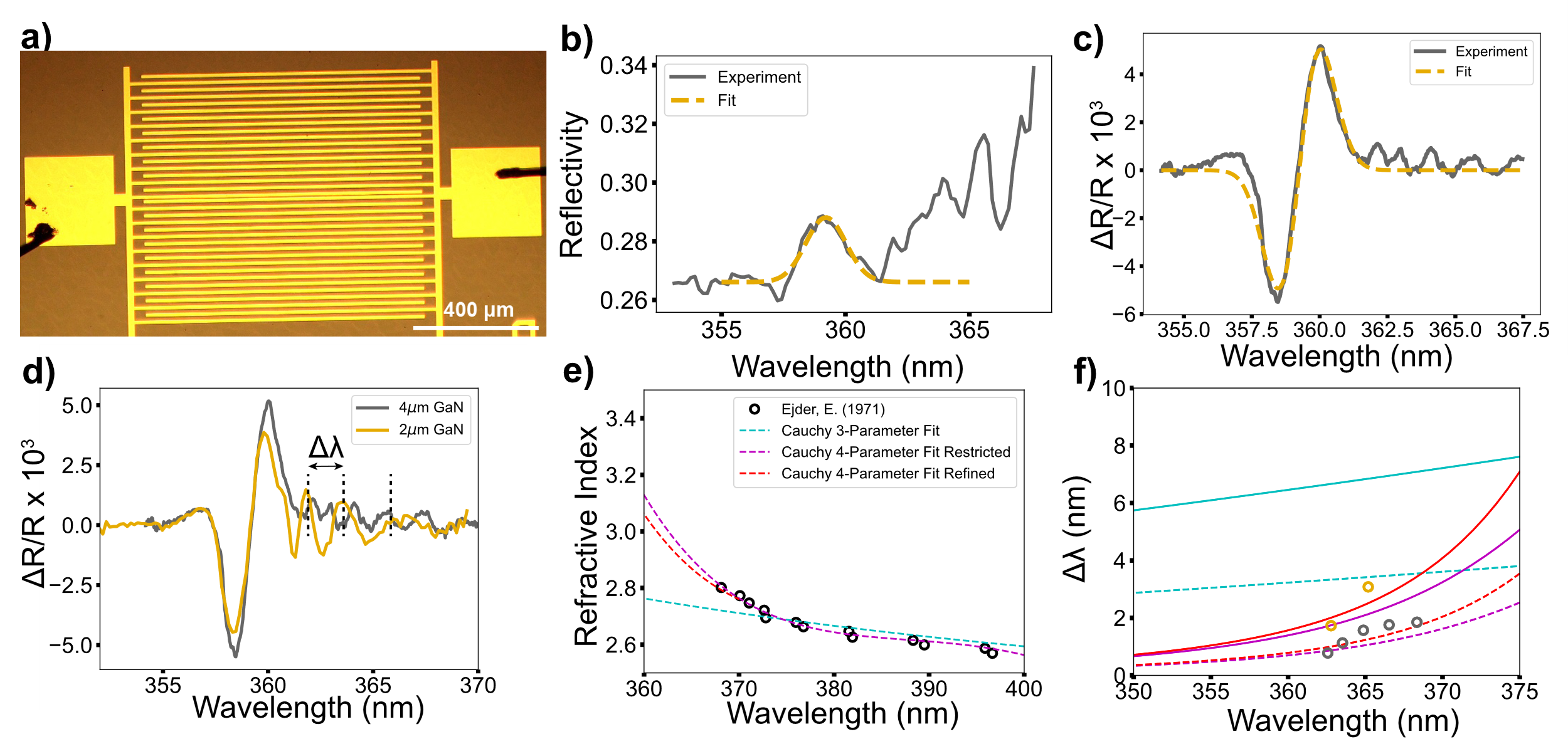


**Figure S1 a)** Microscope image of the device. **b)** Reflectivity spectrum of si-GaN (gray) with a Gaussian fit to the exciton feature (yellow). **c)** Reflection contrast spectrum of GaN with the calculated lineshape using fit routine that considers the redshift of the exciton peak. **d)** Reflection contrast spectrum of 2 and 4 µm thick si-GaN thin film. **e)** The refractive index of GaN (black) with the 3 different extrapolations towards wavelengths of interest. **f)** The fringe spacing extracted from the spectra in **d)** compared to the fringe spacing extracted from the extrapolated refractive indices.

Figure S1b shows the reflectivity spectrum of si-GaN. The exciton peak was fit to a Gaussian using Eq.1.

$$\begin{aligned} R\left( \lambda\right)=ae^{-\frac{\left( \lambda-\lambda_{o} \right)^{2}}{2\Gamma^{2}}}+b\#\left( 1 \right) \end{aligned}$$

A Gaussian with a central energy of 3.451 eV adequately matches the reflectivity spectrum in this wavelength range as shown in Figure S1b. To capture the redshift of the exciton peak position, we use the fit from Figure S1 and slightly adjust the exciton peak position (i.e. $\lambda_{o}+\Delta\lambda$). By employing the shifted and zero field reflectivity, we compute a reflection contrast spectrum. We minimize the difference between the calculated and the experimental reflection contrast by adjusting $\Delta\lambda$ as the only fit parameter. To assess the resilience of the employed fitting routine to experimental variations, we employed a bootstrapping method that generated 1000 electro-optic spectra per electric field by adding Gaussian noise to the experiment data. We chose the spread of the Gaussian noise as 5 times the experimental noise floor and used a Nelder-Mead simplex method to find the line shape that best matched each bootstrapped spectrum. Figure 2a shows the mean of all the bootstrapped lines while Figure 2b presents the standard deviation of these bootstrapped samples as the error bar.

Besides the exciton feature outlined in the main text, we also observe below band gap oscillations in the reflection contrast spectrum that is not captured by the red shift of the exciton. We assign these oscillations to a thin film interference effect by comparing the reflection contrast spectrum measured on a 4 µm thick GaN thin film and a 2 µm one (Figure S1d). The fringe spacing ($\Delta\lambda$) for the thin substrate doubles compared to the thick substrate. The limited number of resolved fringes and the strong dispersion of GaN at these wavelengths prevent a quantitative extraction of the refractive index (*n*) of GaN directly from the fringe spacing. Instead, we use a Cauchy fit to the known refractive index of GaN and calculate the expected fringe spacing. Even this is complicated by the lack of high-quality refractive index data on GaN that spans these wavelengths.

The thin film interference is described by:

$$\begin{aligned} 2d\Delta\beta=2\pi m\#\left( 2 \right) \end{aligned}$$

where *d* is the thin film thickness, *m* is an integer,

$$\begin{aligned} \beta=\frac{2\pi m}{\lambda}\#\left( 3 \right) \end{aligned}$$

The strong dispersion requires refractive index to be described by a model, we choose a Cauchy model with 3 ($n_{0},A,B)$ or 4 ($n_{0},A,B,C)$ parameters.

$$\begin{aligned} n=n_{0}+\frac{A}{\lambda^{2}}+\frac{B}{\lambda^{4}}+\ldots\#\left( 4 \right) \end{aligned}$$

Then using Eq. 3 and 4:

$$\begin{aligned} \frac{d\beta}{d\lambda}=-2\pi\left( \frac{n_{0}}{\lambda^{2}}+\frac{3A}{\lambda^{4}}+\frac{5B}{\lambda^{6}}+\ldots\right)\#\left( 5 \right) \end{aligned}$$

and plugging Eq. 5 into Eq. 2:

$$\begin{aligned} \Delta\lambda=\frac{m}{2d\left( \frac{n_{0}}{\lambda^{2}}+\frac{3A}{\lambda^{4}}+\frac{5B}{\lambda^{6}}+\ldots\right)}\#\left( 6 \right) \end{aligned}$$

We use the refractive index from Ejder^1^ and extrapolate towards the wavelengths of interest using 3 different estimations and extract model parameters ($n_{0},A,B,[C])$. In Figure S1e, we show 3 different extrapolations of the refractive index. Using these models of dispersion and Eq. 7, we calculate the expected fringe spacing for *d* = 2 and *d* = 4 µm separately. In Figure S1f, we show the calculated fringe spacing as the full (*d* = 4 µm) and dashed (*d* = 2 µm) lines. The lines are color coded; they correspond to the refractive index used in the calculation (Figure S1e). We see a qualitative agreement between the experiment results for both *d* = 4 µm and *d* = 2 µm.

SM2-Crystal symmetry constraints on the excitonic Stark effect

In a hydrogen atom, due to the symmetry of the wavefunction, the 1s state exhibits only a quadratic Stark effect, while the 2p state can experience linear Stark effects. In crystals, the crystal field effect can also influence the excitonic Stark effect in a more complicated way. Here, we perform the crystal symmetry analysis of excitonic Stark effects.

Let the static electric field be denoted as $\boldsymbol{F}_{\boldsymbol{1}}=F_{1i}\boldsymbol{e}_{\boldsymbol{i}}$ where the Einstein summation convention is used throughout. The probe field, which measures the reflection spectrum, $\boldsymbol{F}_{\boldsymbol{0}}\left( \omega\right)=F_{0i}\left( \omega\right)\boldsymbol{e}_{\boldsymbol{i}}$, with the assumption ${|\boldsymbol{F}}_{\boldsymbol{1}}\left| \gg{|\boldsymbol{F}}_{\boldsymbol{0}} \right|$. The dielectric function under perturbation of the static electric field can be written as

$$\varepsilon_{ij}\left( \omega\right)=\varepsilon_{ij}^{\left( 0 \right)}\left( \omega\right)+\varepsilon_{ij,k}^{\left( 1 \right)}\left( \omega\right)$$

$$F_{1k}+\varepsilon_{ij,kl}^{\left( 2 \right)}\left( \omega\right)F_{1k}F_{1l}+\ldots$$

The electric displacement field is given by

$$D_{i}\left( \omega\right)=\varepsilon_{ij}\left( \omega\right)F_{0j}\left( \omega\right)$$

We will show that expansion coefficients of $\varepsilon$ are related to the nonlinear susceptibility $\chi$. Noting that the static electric field corresponds to $\omega=0$, the first-order response under the total electric field ($\boldsymbol{F}_{\boldsymbol{1}}\boldsymbol{+}\boldsymbol{F}_{\boldsymbol{0}}$) at finite frequency $\omega$ is

$$P_{i}^{\left( 1 \right)}\left( \omega\right)=\chi_{ij}^{\left( 1 \right)}\left( \omega\right)F_{0j}\left( \omega\right)$$

This leads to the conventional relationship between $\varepsilon$ and $\chi$ in the absence of $\boldsymbol{F}_{\boldsymbol{1}}$

$$\varepsilon_{ij}^{\left( 0 \right)}\left( \omega\right)=1+4\pi\chi_{ij}^{\left( 1 \right)}\left( \omega\right)$$

For GaN, the point group is $C_{6v}$, resulting in $\varepsilon_{xx}^{\left( 0 \right)}=\varepsilon_{yy}^{\left( 0 \right)}$ and $\varepsilon_{xy}^{\left( 0 \right)}=0$.

The second-order response at finite frequency $\omega$ is (the factor 2 comes from exchanging the order of two field)

$$P_{i}^{\left( 2 \right)}\left( \omega\right)={2\chi}_{ijk}^{\left( 2 \right)}\left( \omega,\omega,0 \right)F_{0j}\left( \omega\right)F_{1k}$$

This yields

$$\varepsilon_{ij,k}^{\left( 1 \right)}\left( \omega\right)=8\pi\chi_{ijk}^{\left( 2 \right)}\left( \omega,\omega,0 \right)$$

By examining the symmetry tables^2^, we find that for GaN, all components of $\chi_{ijk}^{\left( 2 \right)}$ in the *x-y* plane are zero: $\chi_{xxx}^{\left( 2 \right)}=\chi_{xxy}^{\left( 2 \right)}=\chi_{xyy}^{\left( 2 \right)}=\ldots=0$, This implies that if both the static electric field and the polarization of the probe field are confined to the *x-y* plane, there will be no first-order correction to the dielectric function in response to the static electric field, i.e., $\varepsilon_{ij,k}^{\left( 1 \right)}\left( \omega\right)=0$ and hence no linear Stark effect.

The third-order response at finite frequency $\omega$ is

$$P_{i}^{\left( 3 \right)}\left( \omega\right)={3\chi}_{ijkl}^{\left( 3 \right)}\left( \omega,\omega,0,0 \right)F_{0j}\left( \omega\right)F_{1k}F_{1l}$$

therefore

$$\varepsilon_{ij,kl}^{\left( 2 \right)}\left( \omega\right)=12\pi\chi_{ijkl}^{\left( 3 \right)}\left( \omega,\omega,0,0 \right)$$

This corresponds to the second-order Stark effect. In GaN, the relevant component are $\chi_{xxxx}^{\left( 3 \right)}=\chi_{yyyy}^{\left( 3 \right)}=\chi_{xxyy}^{\left( 3 \right)}+\chi_{xyyx}^{\left( 3 \right)}+\chi_{xyxy}^{\left( 3 \right)}$, and $\chi_{xxxy}^{\left( 3 \right)}=\chi_{xyyy}^{\left( 3 \right)}=\ldots=0$. When the static electric field and the probe field polarization are aligned and restricted to the *x-y* plane,

$$\boldsymbol{F}_{\boldsymbol{1}}=F_{1}\cos\theta\boldsymbol{e}_{\boldsymbol{x}}\boldsymbol{+}F_{1}\sin\theta\boldsymbol{e}_{\boldsymbol{x}} \boldsymbol{F}_{\boldsymbol{0}}=F_{0}\cos\theta\boldsymbol{e}_{\boldsymbol{x}}\boldsymbol{+}F_{0}\sin\theta\boldsymbol{e}_{\boldsymbol{x}}$$

The third-order polarizability along the *x*-axis is:

$$P_{x}^{\left( 3 \right)}\left( \omega\right)=3F_{0}F_{1}^{2}\left[ \chi_{xxxx}^{\left( 3 \right)}\cos^{3} \theta+\left( \chi_{xxyy}^{\left( 3 \right)}+\chi_{xyyx}^{\left( 3 \right)}+\chi_{xyxy}^{\left( 3 \right)} \right)\sin^{2} \theta\cos\theta\right]$$

$$=3F_{0}F_{1}^{2}\chi_{xxxx}^{\left( 3 \right)}\cos\theta$$

Similarly, along the *y*-axis:

$$P_{y}^{\left( 3 \right)}\left( \omega\right)=3F_{0}F_{1}^{2}\chi_{xxxx}^{\left( 3 \right)}\sin\theta$$

This indicates that the induced polarizability also aligns with the probe field polarization, and $|P^{(3)}\left( \omega\right)|$ is invariant with respect to $\theta$. Consequently, the change in the dielectric constant and the reflection spectrum remains unchanged as long as the static electric field and probe field polarization are aligned and confined to the *x-y* plane.

SM3-Details of the numerical method

First-principles calculations of the electronic structure of GaN (as the mean-field starting point of the GW and BSE) were performed at the DFT-PBE level, as implemented in the Quantum ESPRESSO package^3,4^, using a norm-conserving pseudopotential^5,6^. A plane-wave energy cutoff of 100 Ry and the experimental structure with the lattice constants: *a* = 3.19 Å, *c* = 5.19 Å was used in the calculations. The *GW* (at *G_0_W_0_* level) and *GW*-BSE calculations, for the quasiparticle and optical properties respectively, were performed using the BerkeleyGW package^7^. The dielectric cutoff was set to 40 Ry. A 9 × 9 × 6 grid and 1600 bands were used to calculate the dielectric function and the self-energy corrections. The dynamical screening effect was treated using the Hybertsen−Louie generalized plasmon-pole model^8^. Within the *GW*-BSE calculations, a patched sampling grid around the $\Gamma$ point was used, and the exciton interaction kernel was interpolated from the coarse 9 × 9 × 6 grid to a fine grid equivalent to 48 × 48 × 33 grid in the whole Brillouin zone, using a linear interpolation scheme. Transitions between 3 valence bands and 1 conduction band were considered to ensure convergence of exciton binding energy calculated from the *GW*-BSE results.

The electro-optic effect of GaN is computed by the *ab initio* TD-a*GW* approach, with real-time propagation of the density matrix in the presence of the external light field^9^. In this theoretical framework, the time-dependent interacting density matrix is given by

$$i\hbar\frac{\partial}{\partial t}\rho_{nm,k}\left( t \right)=\left[ H^{aGW}\left( t \right),\rho\left( t \right) \right]_{nm,k}$$

where *n* and *m* are band indices, and $\rho_{nm,k}\left( t \right)$is the interacting density matrix in the Bloch-state basis, which is the key quantity to compute light-induced phenomena. $H_{nm,k}^{aGW}\left( t \right)$ is the TD-a*GW* Hamiltonian, defined as $H_{nm,k}^{aGW}\left( t \right)=h_{nm,k}+U_{nm,k}^{ext}\left( t \right)+\Delta V_{nm,k}^{ee}\left( t \right)$, where $h_{nm,k}$ represents the equilibrium quasi-particle energies, includes all interactions at equilibrium (before the application of an external optical field) at the *GW* level. The external field part $U_{nm,k}^{ext}\left( t \right)$ represents the light-matter interaction and is given by $-e\boldsymbol{E}\left( t \right)\cdot\dot{\boldsymbol{d}_{nm,k}}$, where $\boldsymbol{E}\left( t \right)$ is the optical electric field, and $\boldsymbol{d}_{nm,k}$ is the dipole matrix (i.e., matrix element of the electron position operator $\boldsymbol{r}$), computed using Berry connections with special treatment for the intraband parts ($n=m$) using a local smooth gauge method^9^. In our calculations, a dephasing factor of 5.8 meV is used to simulate the experimental spectral broadening (corresponding to the broadening $\Gamma$ in SM-1). Importantly, excitonic (electron-hole interaction) effects within the TD-a*GW* approach are accurately captured by the photon-field driven time variations in the electron-electron interaction term ${\Delta V}_{nm,k}^{ee}\left( t \right)=\Delta V_{nm,k}^{H}\left( t \right)+\Delta\Sigma_{nm,k}^{COHSEX}\left( t \right)$. Here, the first term represents changes in the Hartree potential, and the second term is the change in the electron self-energy, approximated as the nonlocal Coulomb hole plus screened-exchange (COHSEX) *GW* self-energy in the static limit.

To calculate the absorption spectrum under different static electric fields, a method analogous to a typical "pump-probe" setup was employed^10^. The “pump” field is a function that activates the static electric field $F_{1}$adiabatically, expressed as:

$$E_{pump}\left( t \right)=\left\{ \begin{aligned} 0\left( t<0 \right) \\ F_{1}t/t_{1}\left( 0\leq t\leq t_{1} \right) \\ F_{1}\left( t>t_{1} \right) \end{aligned} \right.$$

The probe field $E_{probe}\left( t \right)=F_{0}e^{-\left( t-t_{0} \right)^{2}/\eta^{2}}$ is applied once the system reaches equilibrium under the static electric field, with ${|F}_{1}\left| \gg{|F}_{0} \right|$. The dielectric function under $F_{1}$ is then calculated from the Fourier transforms of the probe optical field $E_{probe}\left( t \right)$ and its induced polarization $\Delta P\left( t \right)$, as

$$\epsilon\left( \omega\right)=1+4\pi\Delta P\left( \omega\right)/E_{probe}\left( \omega\right)$$

In our TD-a𝐺𝑊 calculations, the time propagation was performed with a time step of 0.0048 fs using a fourth order Runge-Kutta method. The parameters used were $F_{0}=207 V {cm}^{-1}$, $\eta=0.2 \mathrm{fs}$, $t_{1}=960 fs$, $t_{0}=2880 fs,$with the time window for the Fourier transformation being $T=3840 \mathrm{fs}$. Three valence bands and one conduction band were included in the simulations. A patched k-grid sampling around the $\Gamma$-point, equivalent to 48 × 48 × 33 in the whole Brillouin zone, was employed.

To extract the exciton peak shift, the exciton peak in the absorption spectrum was fit using Lorentz broadening:

$$\epsilon_{2}\left( \omega\right)=\frac{A}{\pi}\frac{\Gamma}{\left( \omega-E_{ex} \right)^{2}+\Gamma^{2}}$$

For each static electric field $F_{1}$, this formula was directly used to fit and obtain the exciton energy $E_{ex}$. For example, Figure S2b shows of the fitting for $F_{1}=51.4 kV {cm}^{-1}$, with the corresponding red shift of the exciton peak given by $\Delta E_{ex}=E_{ex,F=51.4 \mathrm{kV}{cm}^{-1}}-E_{ex,F=0}=0.492 \mathrm{meV}$.


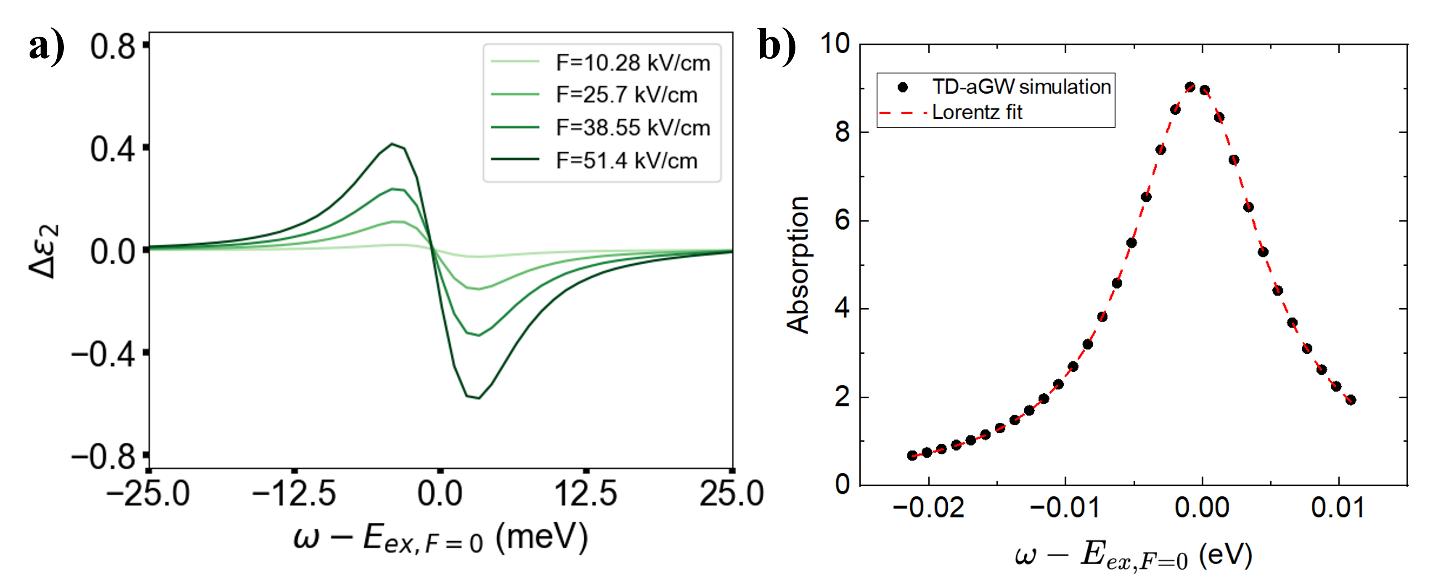


***Figure S2 a)*** *Change in the absorption of the exciton under increasing electric field, showing a redshift of the exciton.* ***b)*** *The black dots are the absorption spectrum of GaN under a static electric field* $F_{1}=51.4 kV/cm$ *calculated by TD-aGW method. The red curve is the Lorentz fitting of the absorption spectrum.* $E_{ex,F=0}$ *denote the exciton energy without the static electric field.*

SM4-Pump-probe curve fitting and distance dependence


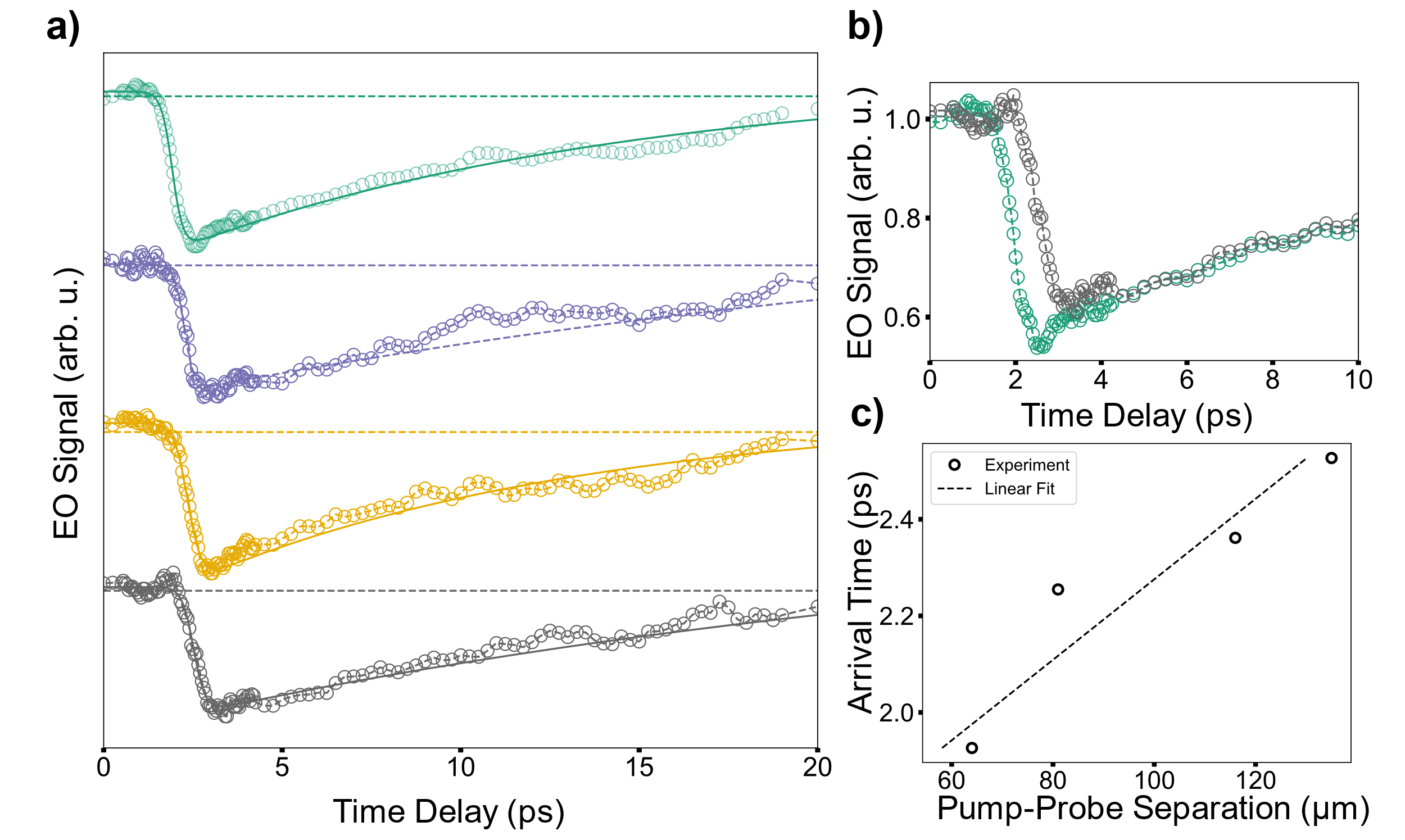


**Figure S3 a)** THz transients as a function of pump-probe separation, traces are offset for clarity. **b)** Two of the traces shown in **a)** without offsets. **c)** The change in the arrival time as a function of pump-probe separation.

V(t) traces shown in the main text Fig. 3b and Fig. 4a are calibrated using a static measurement of the reflectance contrast as a function of electric field. Following arguments regarding the quadratic Stark shift of the exciton energy, we of the reflectance contrast amplitude, however due to the wide bandwidth of the ultrafast probe pulses.

Throughout the manuscript, for quantitative analysis of carrier transit times ($\tau_{t}$) and recovery times ($\tau_{r}$) we use the following equation to fit the observed transients.

$$V\left( t \right)=V_{o}- \Delta V e^{- \frac{\left( t-t_{0} \right)}{\tau_{r}}} erfc\left( -\frac{\left( t-t_{0} \right)}{\tau_{t}} \right) Eq. 8$$

$$erfc\left( x \right)=1-\frac{2}{\sqrt{\pi}}\int_{0}^{x} e^{-t^{2}}dt$$

$V_{o}$ is applied bias, $\Delta V$ is proportional to observed drop around the pulse arrival time $t_{0}$. These fit results are shown with the full lines while the experimental data are the empty circles. The exponential term in Eq. 8 describes the slow carrier recovery, meanwhile error function describes the rapid carrier transit.

As shown in the main text Figure 3a, the pump and probe beams are spatially well separated. The distance between the pump and probe location controls the arrival time of the THz pulse at the probe location. Figure S3a shows results of an experiment where the pump-probe beam separation was varied from 60 to 150 µm. We see a ~0.5 ps shift in the pulse arrival time, extracted as *t*_0_ from the phenomenological fit. In Figure S3b, two traces are overlayed to illustrate the *t*_0_ shift. Figure S3c shows the clear dependence of the pulse arrival time on pump-probe distance. The slope of the dashed line is proportional to the effective refractive index of the waveguided THz mode which is *n*_eff_ = 2.45.


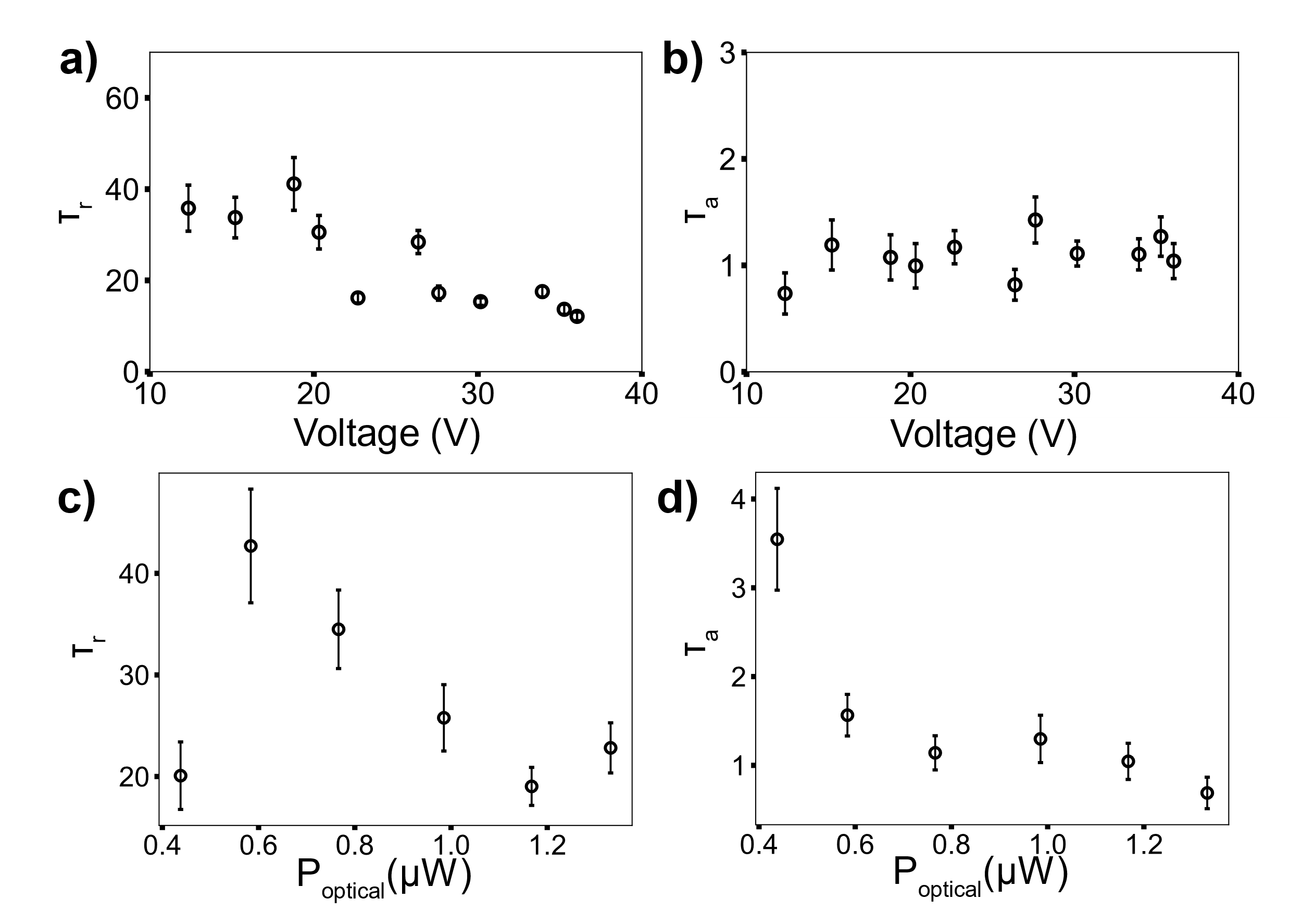
 **Figure S4** Time constants of the voltage dependent experiment from the main text (Figure 3); recombination time is shown in **a)** while the effective transit time is shown in **b)** as a function of bias. Time constants of the intensity dependent experiment from the main text (Figure 4); recombination time is shown in **c)** while the effective transit time is shown in **d)** as a function of intensity.

SM5-Reproducibility of results

Over the course of our study, we developed a library of results that allowed us to focus on the experimental conditions we outline in the main text as the safe operation window where repeated measurements over the course of a week result in reproducible and stable results. In this section we describe a qualitative discussion of factors that we encountered for both the electric field dependent redshift of the exciton feature as well as the terahertz generation results. We also present experimental results from a second device and show the reproducibility of the reported terahertz traces.

1. In terms of reproducibility of the electric field dependent redshift of the exciton feature, we present data from two distinct devices in the supplementary information (SM-1), one device fabricated on a 2 µm thick GaN thin film and one device fabricated on 4µm thick GaN which show similar responses (Figure S1d). We find that the key physical property that determines strength of the excitonic effect is the resistivity of the GaN wafer. In general, low resistivity GaN resulted in the disappearance of the excitonic effect due to free-carrier screening.
2. In terms of reproducibility of the terahertz generation results we found that the dark resistance of devices as well as their photoconductive behavior (such as the one measured in Figure 1 of the main text) are good indicators of terahertz performance. The high-resistivity, semi-insulating GaN wafers results in devices with greater than GΩ dark resistance with photoconductive gain (G) of order 1. We did encounter outlier behavior in some instances. Devices fabricated on the edge pieces of these wafers consistently showed ~10kΩ dark resistance and therefore had a large dark current (leakage current). The large leakage current resulted in devices that did not show any measurable terahertz fields. Further, we had poor electric field breakdown performance from devices that showed high photoconductive gain. This makes intuitive sense considering that a large photoconductive gain is usually attributed to a long carrier recombination time (~on the order of ns to µs, which is far longer than the relevant time scale for terahertz generation ~ps). We hypothesize that the long recombination time results in charge accumulation over multiple laser pulses and that the accumulated charge can lead to damage at the GaN-electrode interfaces. Specifically, we encountered this challenge when exploring passivation strategies of the GaN surface with a thin layer of dielectric. Photoconductivity measurements of these devices resulted in G>1000 presumably due to long-lived charge traps introduced at the GaN-dielectric interface. In these instances, we were not able to push the electric field above few 10’s of kV cm^-1^ and as such we were not able to measure any appreciable terahertz fields.
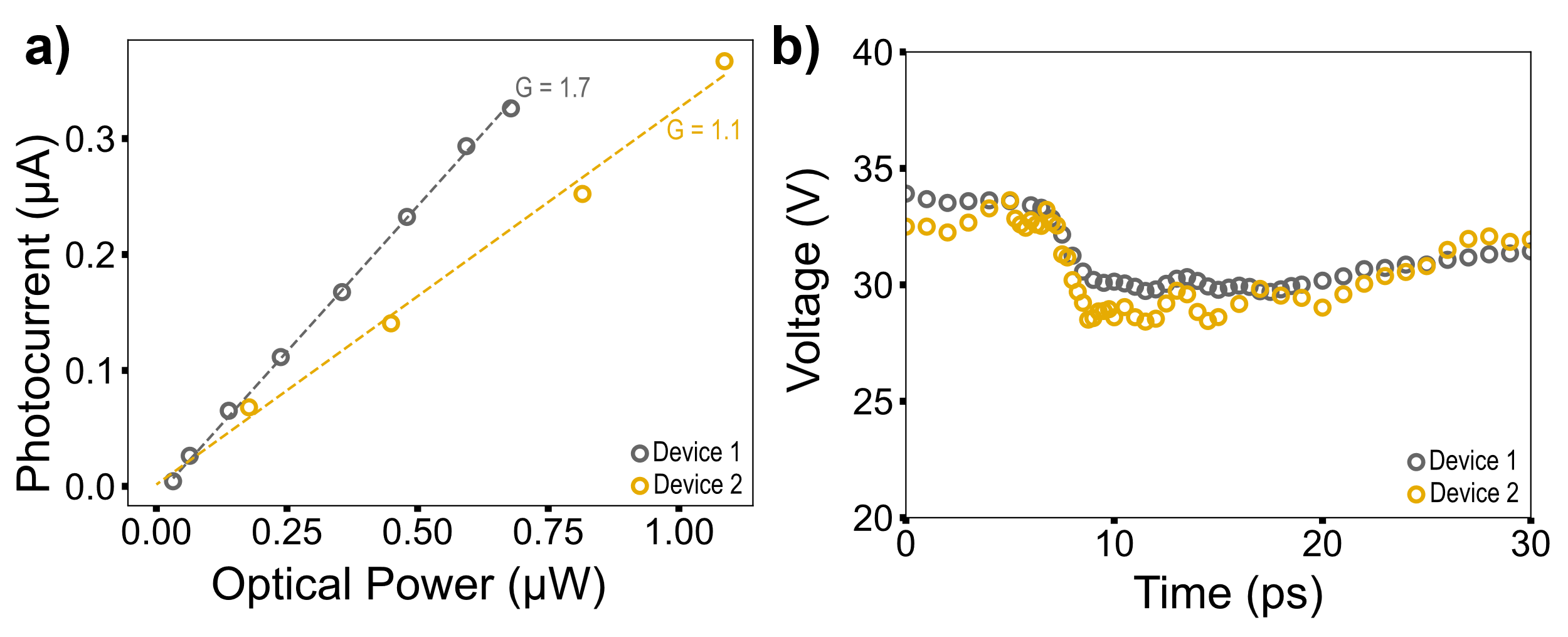


**Figure S5** Results comparison between two separate devices, we label the device from the main text as Device 1 while Device 2 is a similar device. **a)** Photoconductivity as a function of optical power for both devices. For Device-1 we re-plot the data from main text Figure 1-c to compare with Device 2 (yellow) **b)** Voltage-time traces of the two devices at ~33V under P_opt_ = 0.58 µW and P_opt_ = 0.62 µW for Device 1 & 2 respectively.

Overall, we find that the terahertz field measurements of the planar strip-line devices are robust to sample-to-sample variation. To illustrate this point, we compare device characteristics of the device used to produce our principal results (labeled Device 1 in Figure-S5), with a second device (labeled Device 2 in Figure S5). These two devices share the same design shown in Figure S1a and were fabricated simultaneously on si-GaN chips following the procedure outlined in the methods section of the main text. The measurements were conducted on the same setup, but Device 2 was left in storage under ambient conditions for about a month (31 days) prior to measurement.

We see that both devices exhibit G~1 (Figure S5a) and the measured voltage pulses are similar within the error margins of both experiments (Figure S5b). We note that the optical power used for Device 2 was slightly larger (P_opt_ = 0.62 µW) than the power used for Device 1 (P_opt_ = 0.58 µW).


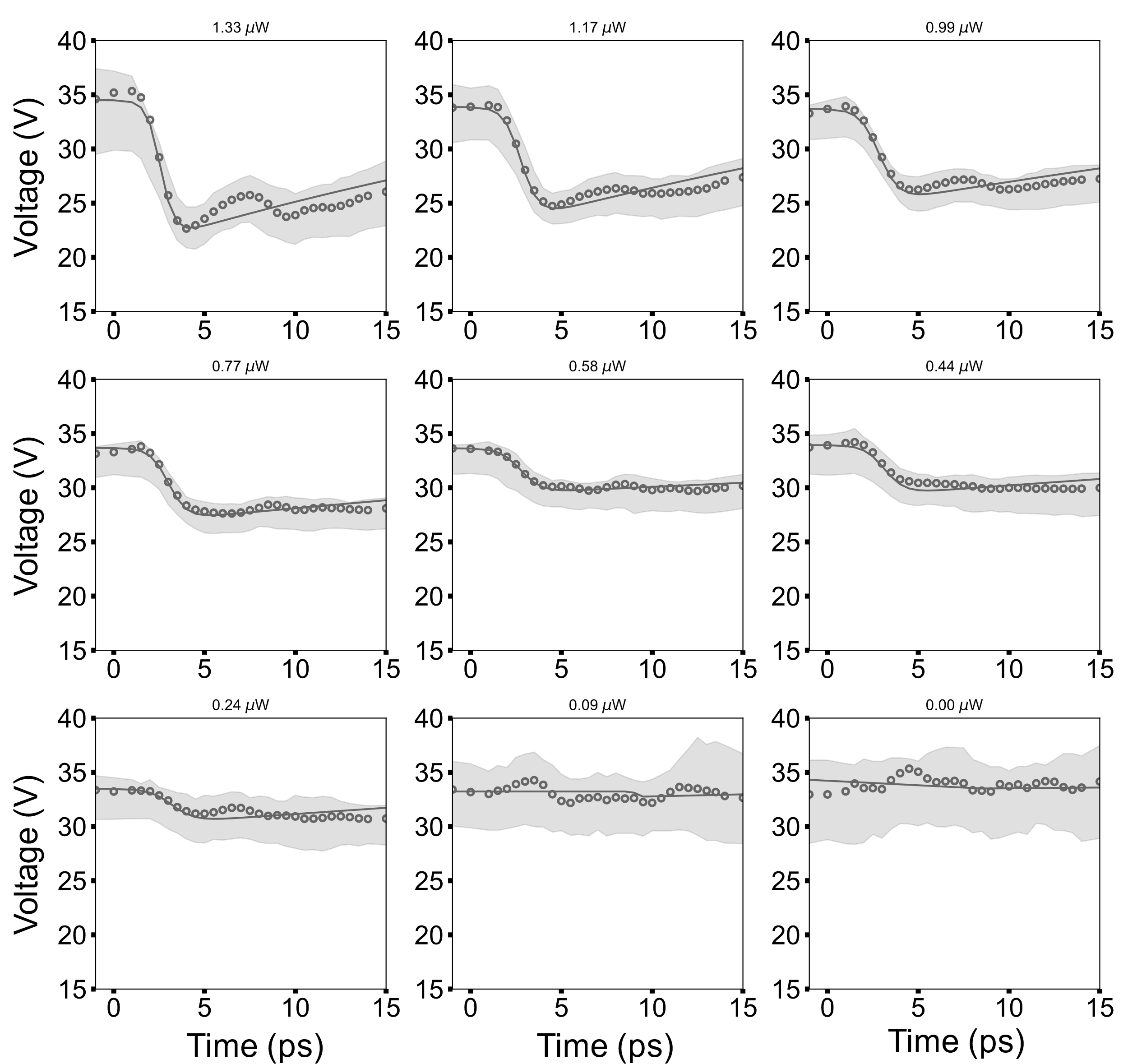


**Figure S6** Each panel corresponds to a voltage-time trace used in the optical power dependent measurements presented in Figure 4a and b of the main text.

SM6-References:

1. Ejder, E. Refractive index of GaN. *physica status solidi (a)* **6**, 445–448 (1971).

2. Boyd, R. W. *Nonlinear Optics*. (Academic Press, Burlington, MA, 2008).

3. Giannozzi, P. *et al.* QUANTUM ESPRESSO: a modular and open-source software project for quantum simulations of materials. *J. Phys.: Condens. Matter* **21**, 395502 (2009).

4. Giannozzi, P. *et al.* Advanced capabilities for materials modelling with Quantum ESPRESSO. *J. Phys.: Condens. Matter* **29**, 465901 (2017).

5. Hamann, D. R. Optimized norm-conserving Vanderbilt pseudopotentials. *Phys. Rev. B* **88**, 085117 (2013).

6. Van Setten, M. J. *et al.* The PseudoDojo: Training and grading a 85 element optimized norm-conserving pseudopotential table. *Computer Physics Communications* **226**, 39–54 (2018).

7. Deslippe, J. *et al.* BerkeleyGW: A massively parallel computer package for the calculation of the quasiparticle and optical properties of materials and nanostructures. *Computer Physics Communications* **183**, 1269–1289 (2012).

8. Hybertsen, M. S. & Louie, S. G. Electron correlation in semiconductors and insulators: Band gaps and quasiparticle energies. *Phys. Rev. B* **34**, 5390–5413 (1986).

9. Chan, Y.-H., Qiu, D. Y., Da Jornada, F. H. & Louie, S. G. Giant exciton-enhanced shift currents and direct current conduction with subbandgap photo excitations produced by many-electron interactions. *Proc. Natl. Acad. Sci. U.S.A.* **118**, e1906938118 (2021).

10. Hu, C., Naik, M. H., Chan, Y.-H. & Louie, S. G. Excitonic Interactions and Mechanism for Ultrafast Interlayer Photoexcited Response in van der Waals Heterostructures. *Phys. Rev. Lett.* **131**, 236904 (2023).
